# Non-Agonistic Bivalent Antibodies That Promote c-MET Degradation and Inhibit Tumor Growth and Others Specific for Tumor Related c-MET

**DOI:** 10.1371/journal.pone.0034658

**Published:** 2012-04-12

**Authors:** Sameer A. Greenall, Ermanno Gherardi, Zhanqi Liu, Jacqueline F. Donoghue, Angela A. Vitali, Qian Li, Roger Murphy, Luisa Iamele, Andrew M. Scott, Terrance G. Johns

**Affiliations:** 1 Oncogenic Signaling Laboratory, Monash Institute of Medical Research, Monash University, Clayton, Victoria, Australia; 2 Medical Research Council Centre, Cambridge, United Kingdom; 3 Tumour Targeting Laboratory, Ludwig Institute for Cancer Research, Heidelberg, Victoria, Australia; Sun Yat-sen University Medical School, China

## Abstract

The c-MET receptor has a function in many human cancers and is a proven therapeutic target. Generating antagonistic or therapeutic monoclonal antibodies (mAbs) targeting c-MET has been difficult because bivalent, intact anti-Met antibodies frequently display agonistic activity, necessitating the use of monovalent antibody fragments for therapy. By using a novel strategy that included immunizing with cells expressing c-MET, we obtained a range of mAbs. These c-MET mAbs were tested for binding specificity and anti-tumor activity using a range of cell-based techniques and *in silico* modeling. The LMH 80 antibody bound an epitope, contained in the small cysteine-rich domain of c-MET (amino acids 519–561), that was preferentially exposed on the c-MET precursor. Since the c-MET precursor is only expressed on the surface of cancer cells and not normal cells, this antibody is potentially tumor specific. An interesting subset of our antibodies displayed profound activities on c-MET internalization and degradation. LMH 87, an antibody binding the loop connecting strands 3d and 4a of the 7-bladed β-propeller domain of c-MET, displayed no intrinsic agonistic activity but promoted receptor internalization and degradation. LMH 87 inhibited HGF/SF-induced migration of SK-OV-3 ovarian carcinoma cells, the proliferation of A549 lung cancer cells and the growth of human U87MG glioma cells in a mouse xenograft model. These results indicate that c-MET antibodies targeting epitopes controlling receptor internalization and degradation provide new ways of controlling c-MET expression and activity and may enable the therapeutic targeting of c-MET by intact, bivalent antibodies.

## Introduction

C-MET, the receptor for hepatocyte growth factor/scatter factor (HGF/SF), is produced as a 170 kDa precursor protein (p170 c-MET) which is subsequently cleaved by the pro-protein convertase furin to produce a disulphide-linked heterodimeric receptor tyrosine kinase (RTK). The mature receptor consists of an extracellular 50 kDa α-chain and an extracellular/intracellular 145 kDa β-chain that contains the TK domain. The α-chain and the N-terminal part of the β-chain associate to form a 7-bladed β-propeller, the SEMA domain, which contains the main binding site for HGF/SF [1]. Upon HGF/SF binding, c-MET homodimerizes leading to activation of its TK domain, as well as autophosphorylation of several tyrosine residues including the C-terminal residues Y1349 and Y1356. Phosphorylated Y1349 and Y1356 form a multi-substrate docking site capable of binding several adaptor proteins to initiate downstream signaling associated with the PI3K/Akt and Ras/MAPK pathways [1], [2].

The HGF/SF:c-MET signaling axis has an important role in the initiation and progression of several aggressive cancers including glioblastoma multiforme (GBM) [3], [4], [5]. As such, c-MET has been intensely investigated as a therapeutic target with several classes of agents being developed as therapeutics, including small molecular weight tyrosine kinase inhibitors (TKIs), which prevent the activation of c-MET by acting as ATP-binding competitors. These TKIs have been shown to have anti-tumor activity in both *in vitro* and *in vivo* models (reviewed in [2], [6]), with several candidates currently being evaluated clinically. Monoclonal antibodies (mAbs) directed to c-MET or HGF/SF represent an alternative class of therapeutics that is attracting considerable interest. Treatment of U87MG GBM xenografts with Rilotumumab, a fully human neutralizing antibody directed to HGF/SF, significantly inhibited tumor growth in mouse xenograft models [7], [8]. Another anti-HGF/SF mAb, TAK-701, effectively reversed c-MET-induced gefitinib resistance in several *in vitro* and *in vivo* models of NSCLC [9].

Antagonistic mAbs directed to c-MET have been difficult to generate as many bivalent antibodies appear to function as agonists. As such, the c-MET antibody in the most advanced clinical trial (MetMAb or Onartuzumab) is a monovalent recombinant antibody fragment derived from an anti-c-MET antibody with agonistic activity [10], [11]. MetMAb appears to function as a classic receptor antagonist by competing with HGF/SF for binding to c-MET [10], [11]. DN-30 is an anti-c-MET antibody with partial agonistic activity [12] that also promotes receptor down-regulation. DN30 was able to inhibit the growth of a gastric cancer xenograft model through stimulating c-MET shedding [13], [14]. Once more, conversion to a monovalent format proved necessary in order to abolish the agonistic activity [15].

Using the human c-MET SEMA domain and live c-MET expressing cells for immunization of mice, we generated a panel of mAbs directed to c-MET which displayed a range of novel properties. These mAbs were assessed biochemically and biologically for their activity on c-MET signalling. The antibodies generated fell into three categories: 1) agonist antibodies as previously reported; 2) a series of antibodies that only bind the c-MET precursor and therefore may be tumor-specific; and, 3) bivalent antibodies that induce c-MET degradation and inhibit tumor growth.

## Results

### Characterization of the LMH anti-c-MET antibody panel

**Figure 1:**
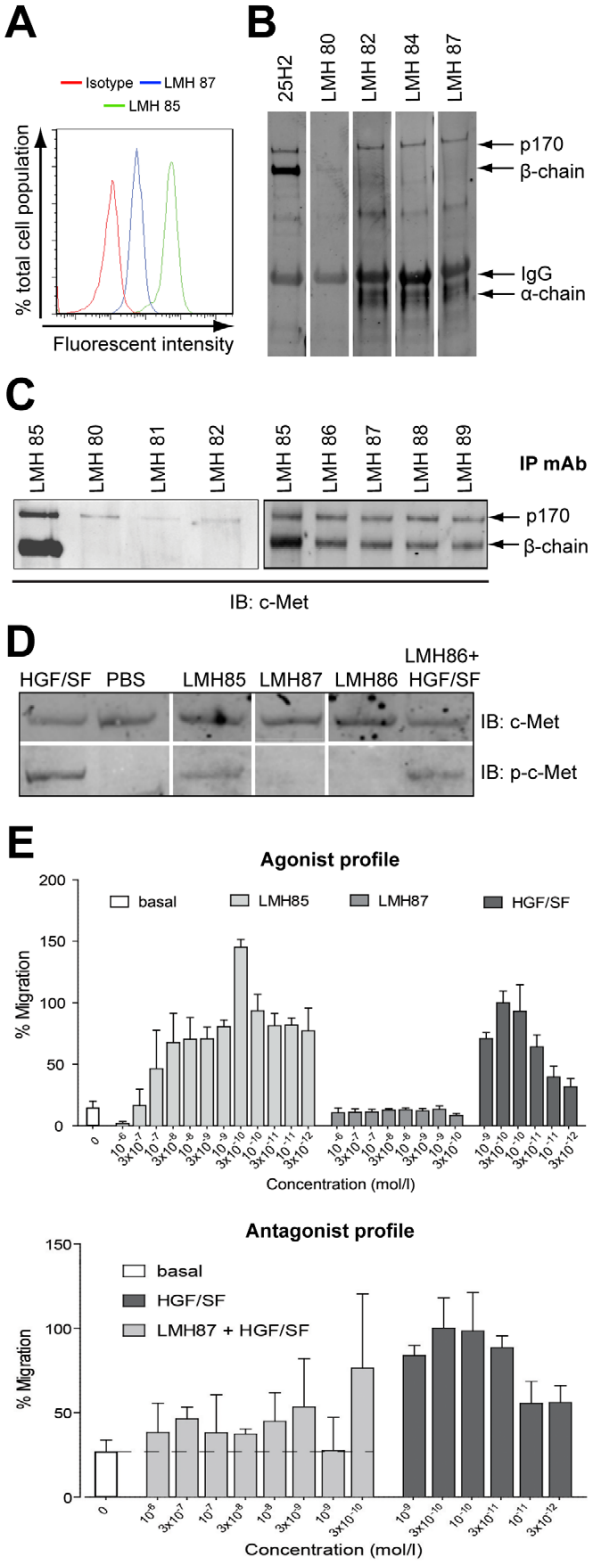
Representative data showing characterization of selected LMH antibodies. (A) flow cytometry analysis showing that LMH 85 and LMH 87 bind surface c-MET expressed on A549 cells. (B) western blots of representative LMH antibodies. c-MET was IPed with LMH 85 and membranes probed with indicated antibodies. The commercial 25H2 recognized the 170 kDa c-MET precursor (p170 c-MET; contains both α and β-chain) and the c-MET β-chain. Using this technique, LMH 80 only weakly bound the β-chain, while LMH 82, 84 and 87 bound p170 c-MET and α-chain. (C) IP with selected LMH antibodies. Following IP with different LMH antibodies, membranes were blotted with mAb 25H2. Antibodies shown were all capable of IP but LMH 80, LMH 81 and LMH 82 appeared specific for p170 c-MET using this technique. (D) biochemical activity of selected LMH antibodies. A459 cells were treated with antibody alone or antibody in the presence of HGF/SF and WCL were probed for total c-MET (*upper panels*) or phosphorylated c-MET (Y1234/Y1235) (*lower panels*). LMH 85 stimulated c-MET phosphorylation, whilst LMH 86 and LMH 87 did not. LMH 86 was unable to block HGF/SF stimulated c-MET phosphorylation. (E) effect of selected LMH antibodies on cell migration. Antibodies were tested for their ability to induce cell migration in SK-OV-3 cells. LMH 85 stimulated cell migration while LMH 87 had no effect (*upper panel*). Differing concentrations of LMH 87 were mixed with 3×10^−10^ M HGF/SF to determine if it inhibited the HGF/SF induced migration of SK-OV-3 cells. LMH 87 substantially inhibited the migratory activity stimulated by HGF/SF (*lower panel*). Data in both graphs are presented as percentage migration versus 3×10^−10^ M HGF/SF ± SD. A full summary of all LMH antibodies is contained in Table 1.

We characterized in detail 10 antibodies (designated LMH) that bound c-MET on the surface of A549 lung cancer cells as determined by FACS (Figure 1A and Table 1). To establish which c-MET chain the antibodies bound, c-MET was immunoprecipitated (IPed) with a commercial pan-c-MET antibody and immunoblotted (IB) with the individual LMH antibodies. The antibody panel contained both α-chain and β-chain binders (Figure 1B and Table 1). Blots for LMH 80 showed it weakly bound the 145 kDa β-chain of c-MET, while LMH 82, LMH 84 and LMH 87 all bound the 170 kDa c-MET precursor (p170 c-MET) and the 50 kDa α-chain of c-MET. Most LMH antibodies were also able to IP c-MET from A549 lysates (Figure 1C and Table 1). Interestingly, LMH 80, LMH 81 and LMH 82 appeared only to IP the p170 c-MET (Figure 1C, *left panel*). The remaining LMH antibodies (Figure 1C, *right panel*), with the exception of LMH 83 that did not IP any c-MET, bound both mature and p170 c-MET.

**Table 1:** Summary of results for a series of biochemical analyses on the properties of the LMH monoclonal antibody panel.

Antibody	ELISA Positive	Flow Cytometry Positive	Immunoblot	IP	Binding Affinity (K_D_)	HGF/SF Antagonist^a^	c-MET Agonist^a^	Antagonist of HGF/SF- Induced Cell Migration^b^	Induces Cell Migration^b^
**LMH 80**	Yes	Yes	Yes, β-chain	Yes, c-MET precursor	34 nM	No	No	n.d.	n.d.
**LMH 81**	Yes	Yes	No	Yes, c-MET precursor	12 nM	No	No	n.d.	n.d.
**LMH 82**	Yes	Yes	Yes, α-chain	Yes, c-MET precursor	n.d.	No	No	n.d.	n.d.
**LMH 83**	Yes	Yes	No	No	26 nM	No	No	n.d.	n.d.
**LMH 84**	Yes	Yes	Yes, α-chain	Yes	36 nM	No	No	n.d.	n.d.
**LMH 85**	Yes	Yes	No	Yes	n.d.	No	Yes	No	Yes
**LMH 86**	Yes	Yes	Yes, α-chain	Yes	n.d.	No	No	No	No
**LMH 87**	Yes	Yes	Yes, α-chain	Yes	2.6 nM	No	No	Yes	No
**LMH 88**	Yes	Yes	Yes, α-chain	Yes	76 nM	No	No	Yes	No
**LMH 89**	Yes	Yes	Yes, α-chain	Yes	31 nM	No	No	Yes	No

n.d. = not determined; IP = immunoprecipitation; HGF/SF = hepatocyte growth factor/scatter factor.

a assays performed using A549 lung adenocarcinoma cells.

b assays performed using SK-OV-3 ovarian carcinoma cells.

As part of our initial screen, all our antibodies were screened biochemically for agonist and antagonist properties using A549 lung cancer cells (Figures 1D–E and Table 1). Incubation of A549 cells with the antibodies in the absence of HGF/SF showed that LMH 85 had agonistic activity (Figure 1D, *bottom panel*), while other antibodies failed to activate c-MET (Table 1). None of the antibodies were able to block short-term, ligand-induced activation of c-MET (see Figure 1D, *bottom panel* for representative results with LMH 86). A sub-set of the LMH antibodies were subsequently screened for biological activity in SK-OV-3 ovarian cancer cells. LMH 85 was able to induce migration in SK-OV-3 cells (Figure 1E, *top panel*), consistent with its ability to induce phosphorylation of c-MET (Figure 1D). In contrast LMH 87 had no effect on cell migration (Figure 1E, *top panel*), consistent with its lack of biochemical agonist activity (Figure 1D). LMH 87 was able to inhibit >75% of HGF/SF-induced migration of SK-OV3 cells at concentrations ≥3×10^−8^ M (Figure 1E, *bottom panel*, *p*<0.05). LMH 88 inhibited HGF/SF-induced SK-OV-3 migration to a similar extent to that observed for LMH 87 (Figure S1B). The biological/biochemical properties of all antibodies, including the binding affinity determined by BIAcore, are summarized in Table 1.

### Single chain variable fragments, made from the agonist LMH 85, are able to block HGF binding to c-MET

**Figure 2:**
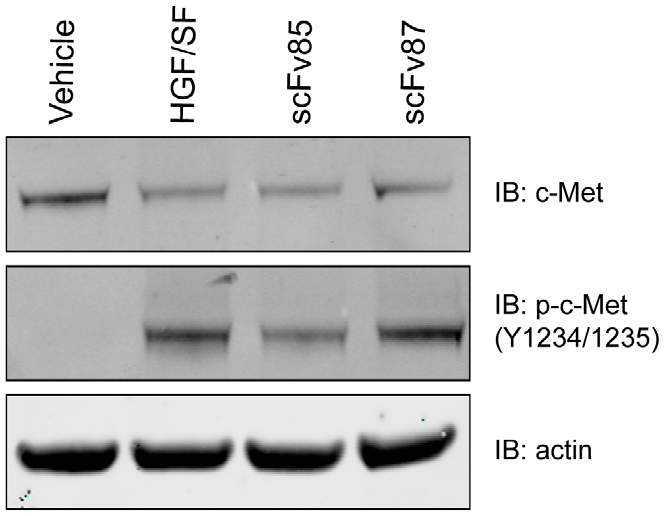
Antagonist activity of single chain variable fragments. LMH 85 and LMH 87 were converted into scFv format and tested for their ability to block HGF/SF stimulation of the c-MET receptor. A549 cells were treated with either scFv 85 or scFv 87 and then HGF/SF added. A vehicle control without HGF/SF was included. Whole cell lysates were then probed for total c-MET (*upper panel*), phosphorylated c-MET (Tyr1234/1235; *middle panel*) and pan-actin to control for loading (*bottom panel*). The phosphorylated c-MET blot shows that scFv 85, but not scFv 87, inhibited HGF/SF stimulation of c-MET.

Single domain antibodies made from anti-c-MET agonist mAbs are sometimes effective in antagonizing HGF/SF induced stimulation of c-MET both *in vitro* and *in vivo* [11]. Therefore we converted the agonist antibody LMH 85, as well as LMH 87, into a single chain variable fragment (scFv) and tested whether they were able to function as a c-MET antagonist. scFv 85 decreased HGF/SF stimulation of c-MET as determined by IB for phosphorylated receptor (Figure 2). In contrast, scFv 87 was unable to block HGF/SF stimulation of c-MET, with levels of phosphorylated c-MET similar to that in the HGF/SF control (Figure 2). Thus, when the agonist mAb LMH 85 was converted to scFv format, it was able to function as a c-MET antagonist.

### Mapping the epitopes of the LMH antibody panel

**Figure 3:**
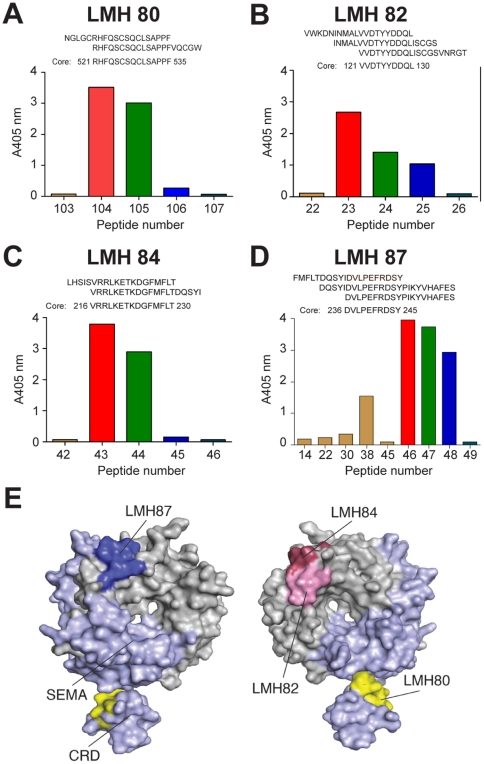
Fine epitope mapping of the LMH antibodies. Antibody reactivity against a panel of overlapping biotinylated peptides representing the c-MET SEMA domain was determined by ELISA. The data shows mean absorbance for selected peptides and the epitope sequences for (A) LMH 80; (B) LMH 82; (C) LMH 84 and (D) LMH 87. (E) epitopes were mapped on to the 3D structure of MET567 incorporating the SEMA domain and cysteine-rich domain (CRD). The areas of the domain corresponding to the α- and β-chains are shown in grey and light blue respectively and the two views are obtained through a 180° rotation along the y axis. Dark blue = LMH 87 (and LMH 86, 88, 89), pink = LMH 82, magenta = LMH 84 and yellow = LMH 80 (and LMH 81). The figure was drawn using PDB accession 1SHY with PYMOL (DeLano Scientific, San Carlos, CA, USA).

To determine the fine epitopes for LMH 80–89, we conducted an analysis with overlapping peptides encompassing the entire c-MET SEMA domain. Most antibodies strongly bound some of the linear peptides with examples of the ELISA results shown in Figures 3A–D. These reactive peptide sequences were then aligned to identify the fine epitope for each antibody (Figures 3A–D). Epitopes were then highlighted within the SEMA domain and cysteine-rich domain (CRD) of MET567 (Figure 3E). LMH 83 and LMH 85 did not bind any linear peptide, indicating that their epitopes are conformational in nature, an observation consistent with the results of IB analyses (Table 1). LMH 86–89 all mapped to an identical DVLPEFRDSY epitope (aa 236–245) within the c-MET α-chain which corresponds to the loop connecting strands 3d-4a on the top side of the c-MET β-propeller (dark blue in Figure 3E, *left panel*). Several antibodies bound to the bottom side of the β-propeller domain including LMH 82 and LMH 84, which mapped to the separate epitopes VVDTYYDDQL (aa 120–129) and VRRLKETKDGFMFLT (aa 215–229), respectively. Both these epitopes are within the c-MET α-chain and contain the loops connecting strands 2a-2b (LMH 82) and strands 3c-3d (LMH 84) (pink and magenta respectively in Figure 3E, *right panel*). Finally, LMH 80 mapped to a sequence (RHFQSCSQCLSAPPFVQCGWC, aa 521–535) within the CRD of c-MET β-chain that contains an 11 amino acid long α-helix (yellow in Figure 3E, *right panel*).

### Docking of LMH 87 onto c-MET

**Figure 4:**
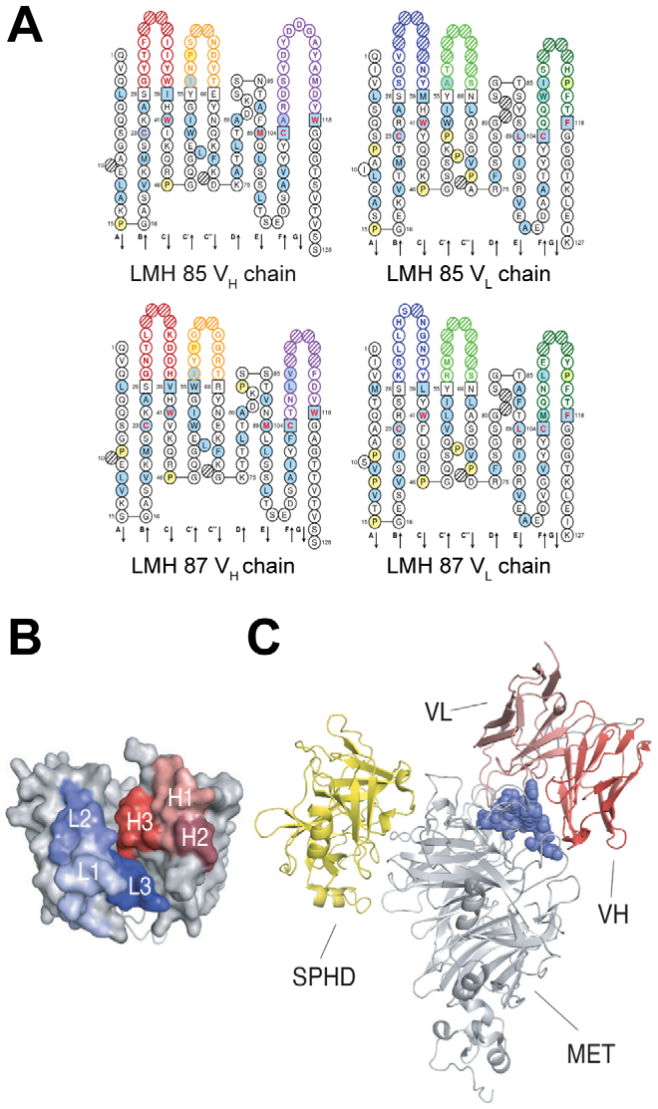
Docking of LMH 87 to c-MET. (A) predicted amino acid sequences of LMH 85 and LMH 87 VH and VL domains. (B) 3D model of the VH and VL domains of LMH87 (modeled as a scFv). The complementarity determining regions (CDR) of the VH and VL domains are shown in red and blue respectively. Framework regions are shown in grey. (C) binding locations within MET567 (grey) of the serine-protease homology domain (SPHD) of HGF/SF (yellow) (PDB accession 1SHY) and the scFv LMH 87 (red). The location of the latter was obtained by docking the model of the LMH 87 scFv onto its epitope (blue spheres). The figure was drawn with PYMOL.

The sequence of the V_H_ and V_L_ chains of LMH 85 and LMH 87 with the predicted amino acid translations are shown in Figure 4A. In order to highlight the binding site of LMH 87, a 3D model of its variable domain was generated (Figure 4B) and the corresponding scFv docked with its epitope within c-MET; an area that includes and surrounds the loop connecting strands 3d-4a (Figure 4C). The binding of LMH 87 was also compared with the binding site of the serine proteinase homology domain (SPHD) of HGF/SF that has been defined crystallographically (Figure 4C) [16]. LMH 87 binds the α-chain of the c-MET β-propeller on its top and side faces, whereas the SPHD domain of HGF/SF binds the α-chain on the bottom face.

### LMH 87 down-regulates total c-MET leading to anti-tumor activity

**Figure 5:**
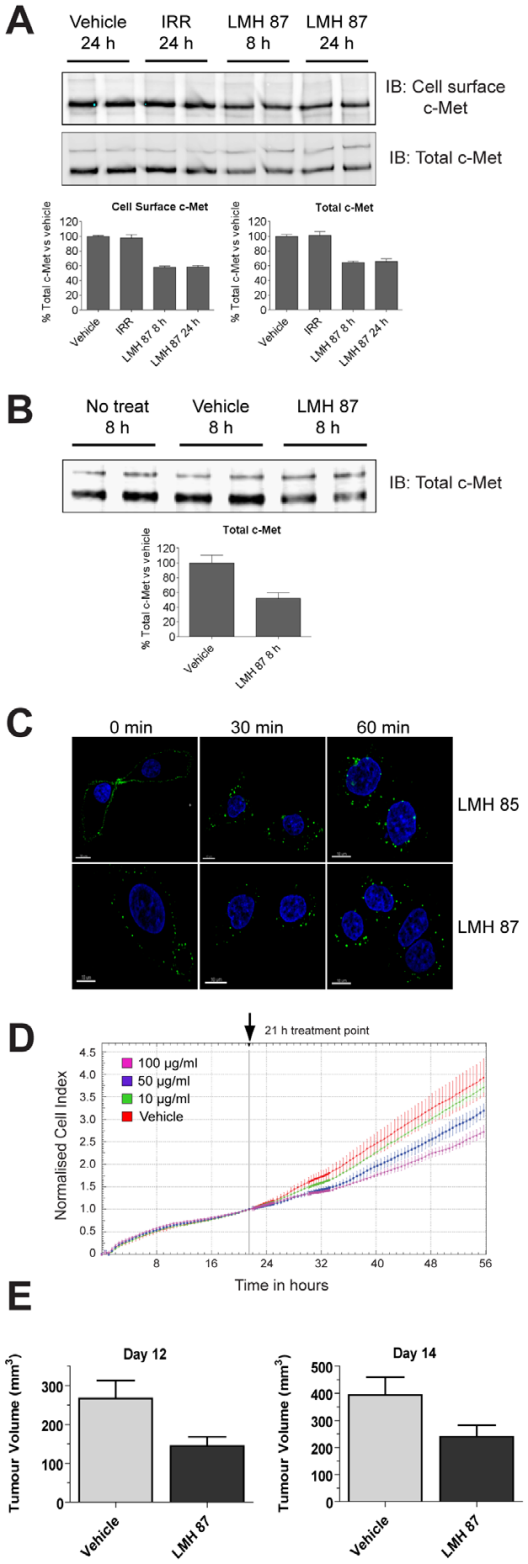
LMH 87 down-regulates surface c-MET and inhibits tumor cell growth. (A) c-MET was IPed using anti-c-MET antibody coupled to agarose beads from A549 lung cancer cells treated with LMH 87 and then cell surface biotinylated. Cell surface c-MET (*upper blot*) and total c-MET (*lower blot*) levels were determined by IB as detailed in the Materials & Methods. Bar graphs show quantification by densitometry ± SEM. IRR = irrelevant antibody. (B) total c-MET remaining in U87MG glioma cells following incubation with LMH 87. Bar graph shows quantification by densitometry ± SEM. Both (A) and (B) are representative blots of repeated experiments. (C) LMH 85 and LMH 87 are internalized following engagement of the c-MET receptor. A549 cells were bound with LMH 85 (*top panels*) or LMH 87 (*bottom panels*) followed by AF488- labeled secondary antibody at 4°C. Internalization was induced with addition of media at 37°C. Significant internalization of both antibodies was observed at 30 min and 60 min as indicated by relocation of the fluorescent signal from the membrane to diffuse cytoplasmic and perinuclear locations. Scale bar = 10 µm. (D) xCELLigence analysis of A549 cells treated with LMH 87. A549 cells were treated with vehicle or different concentrations of LMH 87. Data is presented as the cell index normalized to 21 h ± SEM, the time point when antibody was added. Both the 50 and 100 µg/ml treatments caused a significant decrease (*p*<0.05) in cell index compared to vehicle control. (E) inhibition of U87MG xenograft growth after LMH 87 administration. Mice were implanted with U87MG cells and injected three times (days 7, 10 and 12) with 1 mg of LMH 87 or vehicle. Data is presented as the mean tumor size in mm^3^ ± SEM. LMH 87 significantly inhibited tumor growth at day 12 (*p* = 0.014) and day 14 (*p* = 0.03).

The partial c-MET agonist mAb DN-30 is able to down-regulate total c-MET surface receptor by promoting receptor shedding [13]. Therefore, we tested LMH 87 for its ability to down-regulate c-MET in A549 lung cancer cells by treating them with antibody for 8 or 24 h. LMH 87 was able to down-regulate cell surface c-MET in A549 cells (Figure 5A, *upper blot*) by 40% as determined by densitometry (Figure 5A, *bar graph*) and total cellular c-MET by a similar percentage (Figure 5A, *lower blot* and Figure 5A, *bar graph*). LMH 87 also reduced total c-MET by 50% in U87MG glioma cells (Figure 5B), as determined by densitometry (Figure 5B, *bar graph*). LMH 86, LMH 88 and LMH 89 bind the same core epitope as LMH 87 but LMH 88 was the only other antibody which reduced c-MET levels; like LMH 87, down-regulation was sustained for at least 24 h (Figure S1A).

To examine this further, the ability of LMH 87 to induce internalization of c-MET was analyzed using confocal microscopy (Figure 5C; *bottom panels*). The LMH 85 antibody (Figure 5C, *top panels*) was included as a positive control for both staining and internalization. The staining for both antibodies was membrane bound, focal and punctate at 0 min, with LMH 85 demonstrating more robust staining than LMH 87. Both antibodies showed internalization at 37°C following incubation for 30 min and 60 min; with diffuse cytoplasmic and perinuclear staining observed along with a loss of peripheral membrane staining. Pre-treatment of A549 with TAPI-2, an inhibitor ofADAM-10, an enzyme which initiates presenilin-dependent regulated intramembrane proteolysis (PS-RIP) of c-MET after DN-30 treatment [14], had no effect on LMH 87-induced c-MET down-regulation (Figure S2). This demonstrates that LMH 87 induces c-MET down-regulation and degradation through receptor internalization and not PS-RIP.

To see if this down-regulation had any functional consequence, in addition to its ability to prevent cell migration (Figure 1E), we investigated the effect of LMH 87 treatment on A549 growth using xCELLigence analysis. Treatment of A549 cells with LMH 87 inhibited cellular growth as depicted by the decrease in normalized cell indices for the 50 and 100 µg/ml treatments at all time points (Figure 5D, *p*<0.05). U87MG glioma cells are dependent on the HGF/SF:c-MET signaling axis for *in vivo* growth [8]. Therefore we tested if the LMH 87 antibody had anti-tumor activity against well established U87MG xenografts. After only three injections of 1 mg LMH 87, tumor growth was significantly inhibited at days 12 and 14 post treatment as compared to the PBS control (Figure 5E, *p*<0.05). Hence, LMH 87 is able to inhibit U87MG glioma growth *in vivo*.

### LMH 80 binds cell surface precursor c-MET and is not internalized

**Figure 6:**
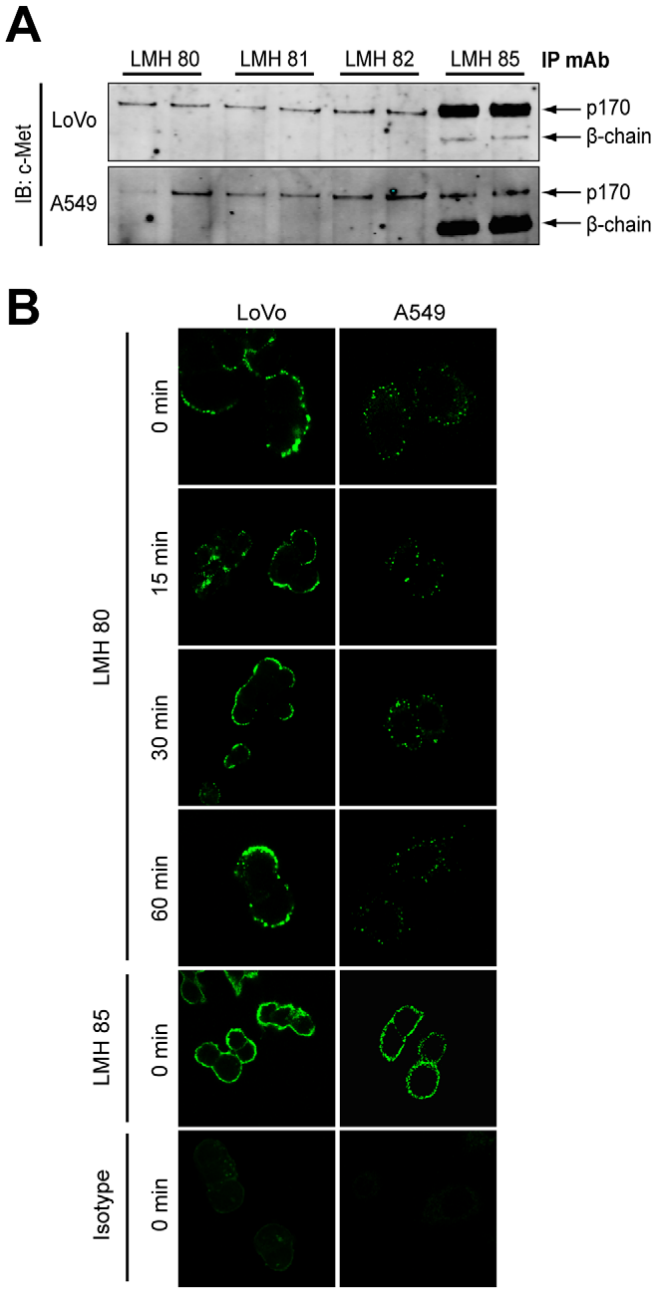
LMH 80, 81 and 82 bind specifically to cell surface p170 c-MET precursor. (A) LMH 80, 81 and 82 only precipitate the p170 c-MET precursor. c-MET was IPed from LoVo cells (*top panel*) or A549 cells (*bottom panel*) using LMH 80, 81, 82 and LMH 85 and probed for total c-MET. A single 170 kDa band was visible in the LMH 80, 81 and 82 lanes, but not the 145 kDa β-chain. The 145 kDa β-chain was clearly present following IP with LMH 85. (B) LMH 80 binds p170 c-MET on the cell surface but is not internalized. Alexa-Fluor 488-labeled LMH 80 was bound to LoVo (*left panels*) and A549 (*right panels*) cells at 4°C to prevent internalization. Warm medium was added to initiate internalization and cells analyzed by confocal microscopy; no significant internalization was observed. A labeled LMH 85 positive control and labeled irrelevant isotype control antibody were included at 0 min.

Our initial experiments suggested that LMH 80, LMH 81 and LMH 82 only bound p170 c-MET (Figure 1C). To evaluate this further we examined these antibodies by IP in A549 lung cancer cells and LoVo colon cancer cells, the latter only expressing the p170 c-MET due to intrinsic defects in c-MET post-translational processing [17]. LMH 80, LMH 81 and LMH 82 specifically bound the p170 c-MET in LoVo (Figure 6A, *top panel*) and A549 (Figure 6A, *lower panel*) cells. While the three antibodies bound all the p170 c-MET in A549 cells, they only IPed a subset of p170 c-MET expressed in LoVo cells. The p170 c-MET was observed on the surface of three different cancer cell lines (A549, LoVo and U87MG) as determined by FACS using all three antibodies (Figure S3). We then tested if LMH 80 is internalized after binding to LoVo and A549 cells by confocal microscopy (Figure 6B). The pattern of staining in both cell lines was focal and punctate. LMH 80 was retained on the cell surface and not internalized for the entire 60 min time course, as illustrated by the lack of cytoplasmic staining in both LoVo (Figure 6B, *upper panels*) and A549 (Figure 6B, *lower panels*) cells. Hence, LMH 80 is able to bind p170 c-MET on the surface of live cells but is not rapidly internalized.

## Discussion

Our investigations have confirmed that anti-c-MET antibodies with receptor antagonistic activity can be generated by converting intact and bivalent antibodies to a monovalent format, a strategy employed in order to generate the MetMAb antibody [10], [11]. This approach also proved necessary in order to remove the partial agonistic activity of the DN-30 antibody [15].

More importantly, the data presented here highlights two novel classes of c-MET-targeting antibodies. The first is the generation of three antibodies (LMH 80–82) that specifically recognize the 170 kDa c-MET precursor at the cell surface. Neither of the two epitopes recognized by these antibodies overlaps with the furin cleavage site itself (i.e. the loop connecting strands 4d and 5a). LMH 80 and LMH 81 bind the α-helix of the CRD while LMH 82 binds the loop connecting strands 2a-2b on the bottom face of the MET β-propeller domain (Figure 3E). This information, and the fact that these antibodies fail to bind mature c-MET by IP, strongly suggest that furin cleavage of the c-MET precursor is accompanied by a major conformational change. Using LMH 80 and non-permeating immunofluorescence, we could definitively establish that p170 c-MET is expressed on cell surface of human cancer cells, in agreement with previous studies in human SkHep1 and LoVo cell lines [17], [18], [19]. Additionally, there is convincing evidence from murine derived cancer cells that p170 c-MET is exposed at the cell surface, and can be activated by HGF [20], [21], [22], [23], [24]. Significantly, non-permeating analyses of primary rat hepatocytes suggested that p170 c-MET was unavailable for ^125^I-HGF/SF binding at the cell surface [25]. The lack of p170 c-MET on the surface of normal cells strongly suggests that its cell surface localization is specific for cancerous cells. The punctate c-MET staining described here for LMH 85 most likely represents non-covalent clusters of c-MET on the cell surface as reported previously [26]. As this pattern was also observed for LMH 80, it indicates that p170 c-MET is probably contained within these non-covalent clusters on the cell surface. LMH 80–82 may be useful for targeting radiotherapeutic and chemotherapeutic agents to tumors while preventing toxic exposure to normal tissues such as liver. Furthermore, as nanoparticle technology develops, these antibodies could be extremely useful tools for promoting the retention of such particles within the tumor mass.

The second novel class of antibody (LMH 87 and LMH 88) is one that promotes c-MET internalization and degradation and/or interferes with receptor recycling to the cell surface (Figure 5A–C). Alterations of RTK degradation and trafficking have emerged in recent years as common and important features of tumor cells as well as a rational target for therapeutic intervention [27]. Binding of the LMH 87 and LMH 88 antibodies to an epitope in the α-chain portion of the c-MET β-propeller promoted receptor degradation independent of HGF/SF, leading to reduced levels of surface c-MET in tumor cells. An initial animal experiment showed that LMH 87 inhibited the growth of U87MG xenografts, confirming that this mAb was effective *in vivo* even in the presence of autocrine HGF/SF. The LMH 87 epitope is distinct from the HGF/SF binding site, suggesting that the intact, bivalent form of this mAb should lack agonistic activity; a hypothesis we confirmed experimentally. Therapeutic inhibition of c-MET with intact, bivalent LMH 87 would have clear advantages over the Fab or scFv formats in terms of stability, half-life and the potential to mediate immune-effector functions. LMH 87's partial antagonistic activity may be advantageous when used in combination with other targeted therapies, especially those targeting EGFR, as it may improve the therapeutic window without significantly increasing toxicity; a potential problem when combining such agents.

The established role of c-MET and HGF/SF in the initiation and progression of a variety of human cancers [1] has stimulated an extraordinary effort into the development of c-MET specific TKIs, with many of these currently under investigation in patients [28]. However, the potential for off-target effects and/or the emergence of resistant populations of tumor cells have already been observed. Thus, despite the difficulties encountered, the drive to develop anti-HGF/SF and anti-c-MET targeted mAbs has a solid and rational foundation [29], [30], [31]. We expect that the two novel classes of anti-c-MET antibodies described here (LMH 80–82 and LMH 87–88) are potential therapeutic candidates for the treatment of human cancer.

## Materials and Methods

### Cells lines and antibodies

The A549, U87MG, LoVo and SK-OV-3 cell lines were obtained from the American Type Culture Collection at low passage number and were all cultured as previously described [3], [8], [32], [33]. Total c-MET antibodies included Met (25H2) (Cell Signaling Technology, Danvers, MA) and Met (c-MET) (Abcam, Cambridge, UK) whilst the phosphorylated c-MET antibody was phospho-Met (3D7) (Tyr1234/1235) (Cell Signaling Technology). Pan actin antibody was Ab-5 (ACTN05) (Thermo Fisher Scientific, Fremont, CA).

### Hybridoma and antibody production

BALB/c mice were immunized four times by i.p. injection at 4-week intervals with 2×10^6^ lysed A549 cells, or c-MET antigen, in incomplete Freund's adjuvant (Sigma) following an initial immunisation in complete Freund's adjuvant (Sigma). Spleen cells were fused with SP2/0 myeloma cells and clonal supernatants were screened by ELISA for c-MET reactivity. Antibody was purified using protein-A affinity chromatography.

### Single chain variable fragment production

BL21.DE3* *E.coli* were transformed with pRSET vector containing the scFv 85 and scFv 87 sequence. After IPTG induction, cell pellets were solublized in 8M urea buffer and purified using Ni-NTA columns. Protein was eluted using a gradient of urea buffer containing increasing concentrations of imidazole. Pooled pure fractions were dialysed with buffer containing 500 mM sodium salt, 1 mM DTT and increasing glycerol/decreasing urea concentrations. The final dialysis was in PBS/5% glycerol.

### Epitope mapping

A PepSet™ Peptide library spanning amino acids 1–567 of the c-MET SEMA domain was synthesized (Mimotopes, Clayton, Australia). The library consisted of N-terminally biotinylated, 20-mer peptides, overlapping by 15 amino acids and pre-absorbed onto 96-well streptavidin coated plates. LMH antibodies 80 to 89 were added to determine their reactivity against each single peptide using standard ELISA.

### Protein modeling and docking

The sequences of the VH and Vk segments of mAb LMH 87 were assembled as scFv's and used for homology modeling using the 3D-Jigsaw server [34]. Models were evaluated using the Ramachandran plot and VERIFY-3D [35] and used for protein-protein docking using the FireDock server [36]. Docking solutions were inspected visually and protein interfaces analyzed using the PISA server [37].

### Surface plasmon resonance

Surface plasmon resonance (BIAcore) was conducted essentially as previously described [38] utilizing immobilized c-MET extracellular domain and different concentrations of purified LMH antibodies to determine *K*_D_ values.

### Flow cytometry

A549 cells were stained for c-MET using 10 µg/ml of each LMH antibody diluted in 1% HSA/PBS for 1 h at 4°C. A 1∶20 secondary anti-mouse IgG-PE (Invitrogen) solution was then added for 45 min at 4°C. Cells were run on a BD CANTO Flow Cytometer (BD Biosciences, San Jose, CA) for analysis.

### Immunoprecipitation, whole cell lysate and western blot

For agonist and antagonist assays, 50 µg/ml of antibody was added to serum starved cells for 30 min. Afterward, for antagonist tests only, 400 ng/ml of HGF was added and cells incubated for a further 7 min. For c-MET down-regulation assays, 50 µg/ml of antibody was added and cells incubated for 8 h or 24 h. For immunoprecipitation (IP) of c-MET, cells were lysed in Triton X-100 cell lysis buffer and processed as previously described [8], using either anti-c-MET C-28 antibody coupled to agarose beads (Santa Cruz) or 5 µg/ml LMH 85 followed by Protein A/G agarose beads. For whole cell lysate (WCL) analyses, cells were scraped in the presence of lysis buffer [1×Invitrogen LDS sample buffer, 2.5% (v/v) β-mercaptoethanol, 200 µM Na_3_VO_4_ and 0.4% (v/v) H_2_O_2_], aspirated through a syringe, sonicated and clarified by centrifugation. Western blots were conducted as previously described [8] using appropriate antibodies for blotting and infrared detection.

### Cell surface biotinylation

A549 cells were plated overnight and washed twice in serum free medium before 24 h treatments in serum free medium were added. The next day, antibody was added to the 8 h treatments using the medium from the dish. At treatment completion, dishes were washed three times with excess PBS at pH 8.0. Next, 0.5 mg/ml of non-permeating EZ-Link® Sulfo-NHS-LC-Biotin (Thermo Scientific) in PBS (pH 8.0) was added and the cells incubated on a rocking platform at 4°C for 1 h. Cells were then quenched three times in excess glycine (100 mM). Cells were lysed and c-MET IPed using anti-c-MET C-28 antibody coupled to agarose beads as described above. Following SDS-PAGE and transfer, membranes were probed for biotinylated c-MET using Streptavidin conjugated to IRDye 680 (LiCor Biosciences) or total c-MET using rabbit anti-Met (1000–1100) polyclonal antibody (Abcam) followed by anti-rabbit IRDye800 secondary antibody. Blots were analysed using infrared detection.

### Cell migration assay

For agonist tests, the bottom wells of the 96-well chemotaxis chambers coated with collagen (Neuro Probe, Gaithersburg, MD) and filled with different antibody concentrations in RPMI/0.25% BSA. For antagonist tests, 0.3 pM HGF was mixed with the antibody. The top of the chamber was filled with 4×10^5^ SK-OV-3 cells in RPMI/0.25% BSA. After 4 h incubation, the membrane was recovered and migrated cells fixed in 4% formaldehyde for 1 h, washed in PBS and stained overnight with DeepRed cytoplasmic stain (Invitrogen). The membranes were scanned using a LiCor Odyssey Infrared scanner at 700 nm to quantify stained cells.

### xCELLigence

A549 cells were plated in 0.5% FBS media in untreated E-plates (Roche Diagnostics, Basel, Switzerland) at 5,000 cells per well. The plate was connected to an xCELLigence RTCA SP instrument (Roche Diagnostics) within a humidified cell culture incubator. Treatments were initiated after 21 h incubation. Data was analysed using the RTCA Software 1.2 program (Roche Diagnostics). Readings were normalized to the point directly before antibody addition. All data is presented as the mean normalized cellular index ± SEM over time.

### Confocal microscopy

For LMH 87 internalization, A549 cells were plated in iBidi 8-well chamber slides overnight. The next day, cells were washed in 1% HSA/serum free medium before 20 µg/mL of antibody was added for 45 min at 4°C, followed by an equimolar amount of anti-mouse IgG labeled with Alexa Fluor 488 (Invitrogen) for 45 min at 4°C. An LMH 85 parallel control was included. For LMH 80 internalization tests, LMH 80 and LMH 85 were directly labeled with Alexa Fluor 488 overnight using the Alexa Fluor 488 Zenon ® Mouse IgG labeling kit (Invitrogen). A549 or LoVo cells were plated on coverslips overnight and washed in 1% HSA/serum free medium before 10 µg/ml of labeled antibodies were added for 45 min at 4°C. To induce internalization in both tests, serum free medium 37°C was added for 0, 15, 30 or 60 min, after which cells were fixed in 4% paraformaldehyde, mounted and scanned using a Nikon C1 confocal microscope equipped with a 60× objective.

### Xenograft model

U87MG xenograft trials were conducted essentially as previously described [8]. Briefly, 1×10^6^ cells were injected into the ventral left and right flanks of 4- to 6-week old female BALB/c nude mice. Treatment was initiated when tumor sizes reached 80–200 mm^3^. An intraperitoneal (i.p.) route of administration was used after which tumor measurement and statistical analyses were performed as previously described [8]. This project was approved by the Monash University Animal Ethics Committee.

## Supporting Information

Figure S1 **LMH 88- induced c-Met degradation and antagonism of HGF/SF-induced cell migration.** (A) U87MG cells were treated with LMH 88 for the indicated times and levels of total c-Met determined by immunoblotting. A sustained decrease in total c-Met levels up to 24 h was observed. (B) SKOV-3 cells were treated with media alone (basal control; white), different concentrations of LMH 88 with 3×10^−10^ M of HGF/SF (light grey) or different concentrations of HGF/SF alone (dark grey) to determine if LMH 88 could inhibit HGF/SF induced cell migration. LMH 88 inhibited the migratory activity stimulated by HGF/SF to a degree similar to that for LMH 87. Data is presented as percentage migration compared to 3×10^−10^ M HGF/SF ± SD.
(TIF)

Figure S2 **LMH 87-induced c-Met down-regulation does not utilise the presenilin-dependent regulated intramembrane proteolysis (PS-RIP) mechanism.** A549 cells were pre-treated with 25 µM of the metalloproteinase inhibitor, TAPI-2, for 30 min before 50 µg/mL of LMH 87 was added for 8 h. Levels of total c-Met were determined by IP and immunoblotting. The results show that inhibition of metalloproteinase, which is critical to initiate the PS-RIP mechanism, had no effect on LMH 87-induced c-Met down-regulation.
(TIF)

Figure S3 **LMH 80, LMH 81 and LMH 82 all bind the cell surface by FACS.** FACS with isotype control antibody or the LMH antibodies was conducted on A549, LoVo and U87MG cell lines. Positive binding of all antibodies confirms that the p170 c-Met is located at the cell surface in cancer cells.
(TIF)
