# Supplementary figures and images for: Non-Agonistic Bivalent Antibodies That Promote c-MET Degradation and Inhibit Tumor Growth and Others Specific for Tumor Related c-MET
Source: PLoS One. 2012 Apr 12;7(4):e34658. doi: 10.1371/journal.pone.0034658 (PMC3325269; doi:10.1371/journal.pone.0034658)

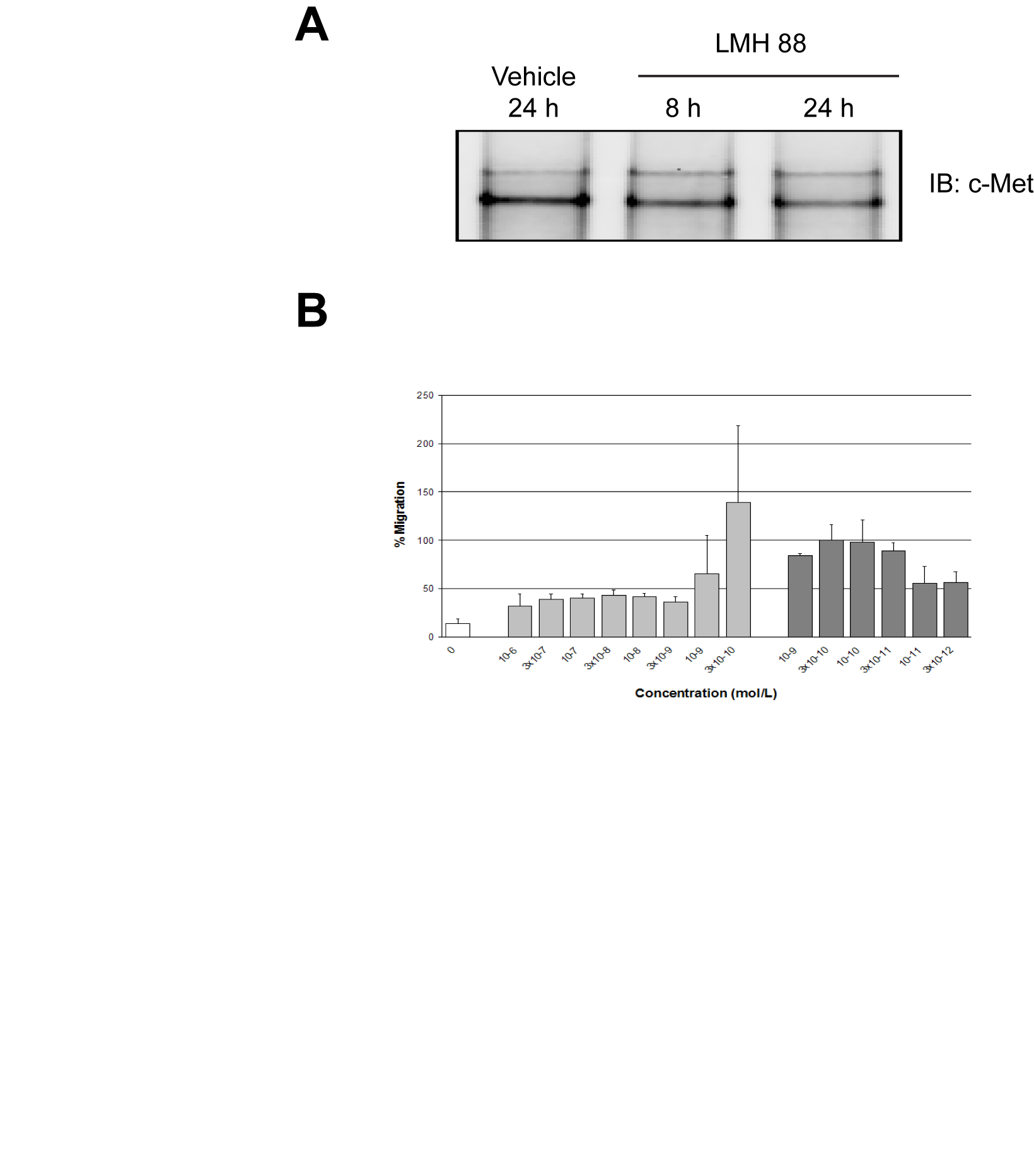

Supplement: Figure S1 — LMH 88- induced c-Met degradation and antagonism of HGF/SF-induced cell migration. (A) U87MG cells were treated with LMH 88 for the indicated times and levels of total c-Met determined by immunoblotting. A sustained decrease in total c-Met levels up to 24 h was observed. (B) SKOV-3 cells were treated with media alone (basal control; white), different concentrations of LMH 88 with 3×10^−10^ M of HGF/SF (light grey) or different concentrations of HGF/SF alone (dark grey) to determine if LMH 88 could inhibit HGF/SF induced cell migration. LMH 88 inhibited the migratory activity stimulated by HGF/SF to a degree similar to that for LMH 87. Data is presented as percentage migration compared to 3×10^−10^ M HGF/SF ± SD. (TIF) [file pone.0034658.s001.tif]

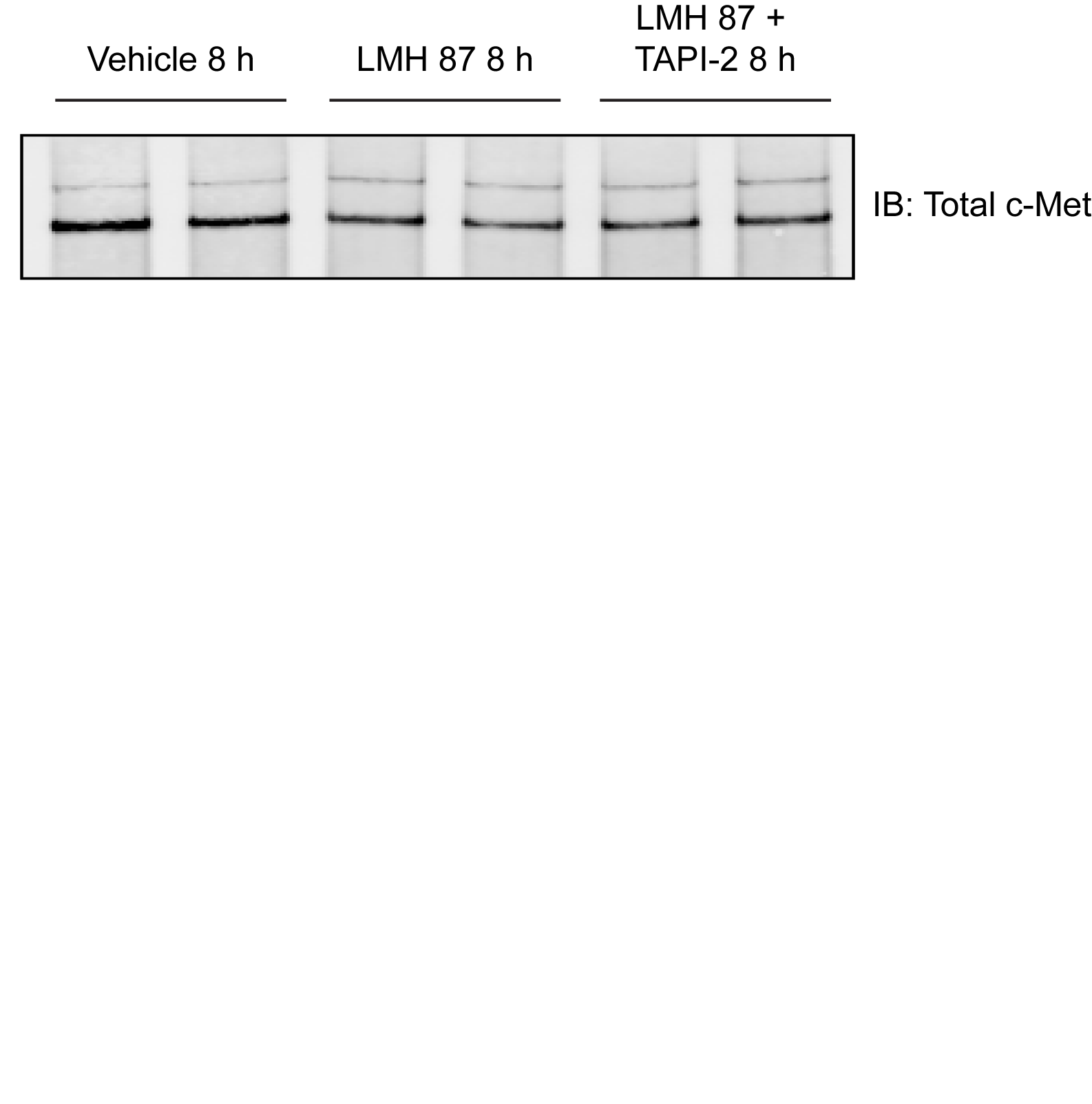

Supplement: Figure S2 — LMH 87-induced c-Met down-regulation does not utilise the presenilin-dependent regulated intramembrane proteolysis (PS-RIP) mechanism. A549 cells were pre-treated with 25 µM of the metalloproteinase inhibitor, TAPI-2, for 30 min before 50 µg/mL of LMH 87 was added for 8 h. Levels of total c-Met were determined by IP and immunoblotting. The results show that inhibition of metalloproteinase, which is critical to initiate the PS-RIP mechanism, had no effect on LMH 87-induced c-Met down-regulation. (TIF) [file pone.0034658.s002.tif]

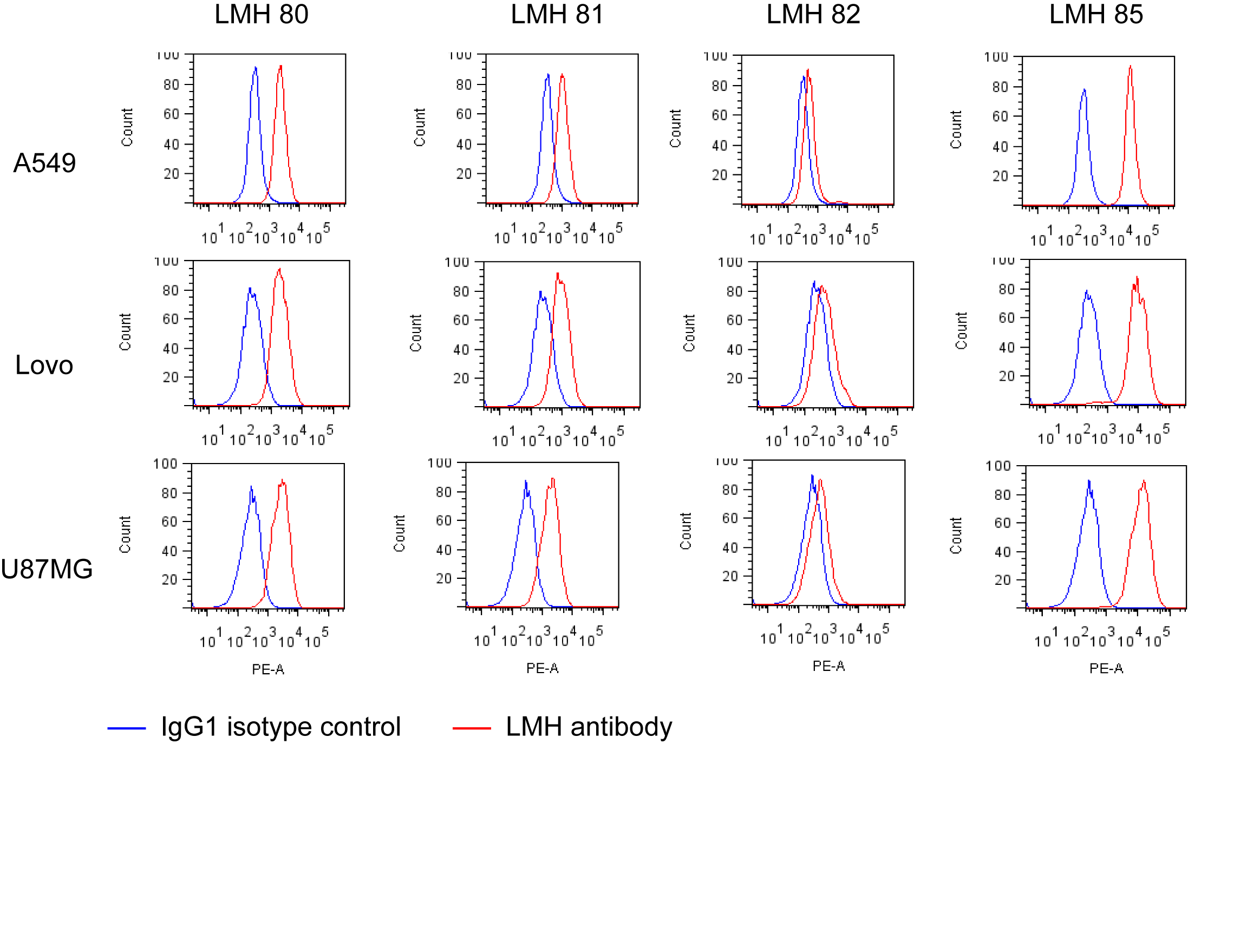

Supplement: Figure S3 — LMH 80, LMH 81 and LMH 82 all bind the cell surface by FACS. FACS with isotype control antibody or the LMH antibodies was conducted on A549, LoVo and U87MG cell lines. Positive binding of all antibodies confirms that the p170 c-Met is located at the cell surface in cancer cells. (TIF) [file pone.0034658.s003.tif]
